# A Boy With KIF11-Associated Disorder Along With ADHD and ASD: Collaboration Between Paediatrics and Child Psychiatry

**DOI:** 10.1155/2024/5535830

**Published:** 2024-09-25

**Authors:** Annelien Marcelis, Evelyne Van Reet

**Affiliations:** ^1^ AZ Sint-Maria Halle, Halle, Belgium; ^2^ Vrije Universiteit Brussel, Brussels, Belgium

**Keywords:** attention deficit disorder with hyperactivity, autism spectrum disorder, case report, chorioretinal dysplasia microcephaly mental retardation syndrome, KIF11 protein, lymphoedema, microcephaly

## Abstract

Kinesin family member 11 (KIF11)-associated disorder, a rare condition caused by autosomal dominant mutations in the KIF11 gene, presents with microcephaly, chorioretinal dysplasia, lymphoedema, and varying degrees of intellectual disability. While intellectual disability is often described in the literature on KIF11 mutations, autism spectrum disorder (ASD) and attention-deficit/hyperactivity disorder (ADHD) are only mentioned by a few authors but not thoroughly investigated. We present a case report of an 8-year-old boy with KIF11-associated disorder alongside ADHD and ASD but without intellectual disability. Genetic testing confirmed a KIF11 mutation. Cognitive, language, and motor assessments revealed delays in fine motor skills and attention deficits. The diagnosis of ADHD was confirmed by a child neurologist through multidisciplinary investigations, while the ASD diagnosis was established by a child psychiatrist. Despite the challenges of delayed psychiatric assessment, interventions including physiotherapy and medication management were initiated with positive results. We designed a parent support group survey that showed a higher prevalence of neurodevelopmental disorders in children with KIF11 mutations compared to the general population. Therefore, low-threshold referrals to a child psychiatrist have to be made when the potential presence of developmental problems is suspected. Collaboration between ophthalmologists, paediatricians, and child psychiatrists is crucial for early detection and intervention. Addressing developmental disorders promptly improves long-term outcomes and enhances quality of life. Moreover, gaining a deeper understanding of the higher prevalence of ASD and ADHD in individuals with KIF11 mutations could offer valuable insights into the genetic mechanisms underlying neurodevelopmental disorders.

## 1. Introduction

A rare condition known as kinesin family member 11 (KIF11)-associated disorder, with a prevalence lower than 1 in 1,000,000, is characterised by varying expressions of microcephaly, either with or without chorioretinal dysplasia, along with lymphoedema and learning disabilities. It is caused by an autosomal dominant mutation in the KIF11 gene [1–5], located on chromosome 10, which encodes for EG5, a motor protein in the kinesin-like protein family. Although the precise function of this gene remains unknown, it appears to play a role in the development of the eyes, lymphatic system, and brain [2]. The diagnosis is made based on genetic testing due to the variable occurrence of microcephaly, ocular abnormalities, lymphoedema, and/or mental retardation.

Neurodevelopmental disorders are commonly associated with KIF11 mutations[3, 6], but no prior research has explored the connection between KIF11-associated disorder and mental disorders. Schlögel et al. [3] reported intellectual disability in 67% of cases, while Jones et al. noted mild to moderate learning disabilities in 73% of cases [6]. On the other hand, conditions like autism spectrum disorder (ASD) or attention-deficit/hyperactivity disorder (ADHD) are occasionally mentioned but not thoroughly investigated [6]. This is likely attributed, at least in part, to the variability in the expression of the disorder. This variability implies that individuals within the same family carrying the same mutation may display diverse phenotypes, a phenomenon extensively documented in various research studies [2–6].

ASD and ADHD are neurodevelopmental disorders that impact behaviour, communication, and learning. Both ADHD and ASD have high heritability. Research indicates a notable overlap between these conditions, with 20%–50% of children with ADHD also meeting the criteria for ASD and 30%–80% of children with ASD meeting the criteria for ADHD [7].

ASD is a neurodevelopmental condition characterised by challenges in social communication and interaction, along with restricted, repetitive behaviours and interests [8]. The aetiology of ASD is likely multifactorial, involving both genetic and environmental factors. ASD can be classified as either syndromic or nonsyndromic. Syndromic ASD is often linked to chromosomal abnormalities or monogenic mutations, as seen in conditions like Rett syndrome. In contrast, the aetiology of nonsyndromic ASD remains less defined due to its genetic complexity. A combination of de novo mutations and prenatal and postnatal environmental factors is thought to contribute to its development [9].

ADHD is a neurodevelopmental disorder marked by persistent patterns of inattention, hyperactivity, and impulsivity [8]. Individuals with ADHD may struggle with staying focused, organising tasks, and following through on instructions, leading to challenges in school, work, and daily life. Hyperactivity can manifest as excessive fidgeting, talking, or an inability to stay still, while impulsivity may result in hasty decisions or interrupting others. Similar to ASD, the aetiology of ADHD involves a combination of genetic and environmental factors [10].

In this case report, we present the profile of a boy affected by KIF11-associated disorder, alongside these two additional neurodevelopmental disorders: ADHD and ASD.

## 2. Case Presentation

### 2.1. Medical

#### 2.1.1. Case History

The case involves an 8-year-old Belgian Caucasian boy, born at 38 weeks to non-consanguineous parents as their first child.

#### 2.1.2. Methods

**Figure 1:**
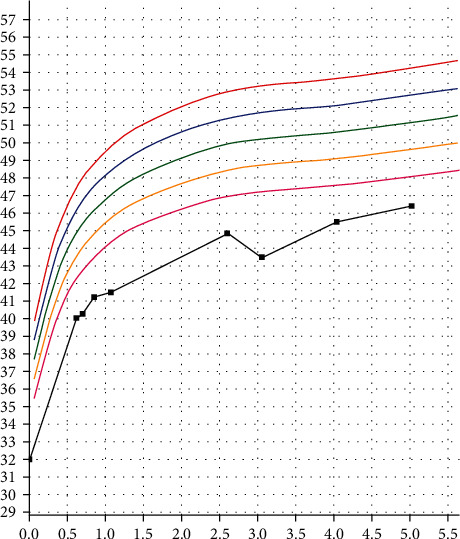
Growth curve with microcephaly. (*x*-axis: age in years, *y*-axis: head circumference in centimetres).

**Figure 2:**
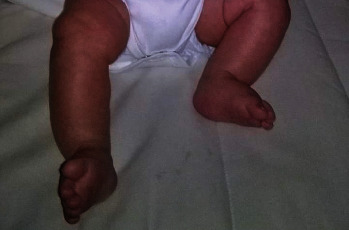
Oedematous feet at birth, retrospectively presumed to be due to lymphoedema.

Amniocentesis, conducted due to an abnormal pregnancy-associated plasma protein-A (PAPP-A) test, yielded normal results. Despite a noticeable microcephaly (Figure 1), ultrasound at 2 months and brain magnetic resonance imaging (MRI) at 10 months both showed normal findings. A microarray indicated a normal molecular karyotype. At the age of 13 months, the recurrent otitis was addressed through the placement of tympanostomy tubes. A screening ophthalmological exam revealed significant myopia and chorioretinal dysplasia. In retrospect, the boy exhibited oedematous feet at birth, likely due to lymphoedema (Figure 2). Due to the ocular abnormalities, the boy was referred by the ophthalmologist for genetic testing.

#### 2.1.3. Outcome and Follow-Up

Genetic testing identified a mutation in the KIF11 gene (c.1791dupT) p.Thr598Tyrfs ^*∗*^8. Current annual follow-up indicates stable vision. In recent months, the boy has been wearing glasses for myopia. The swelling in the feet spontaneously resolved when the boy began crawling. Persistent toilet training difficulties necessitate hospital admissions for colon cleansing and pelvic floor physiotherapy, along with intake of macrogol 4000 10 g once daily and rectal glycerin suppository every 2 days.

### 2.2. Psychiatric

#### 2.2.1. Case History

At the age of 3, parents first expressed concerns that their son struggled to follow instructions, required repeated instructions, and displayed restlessness. While his motor and language development progressed without significant problems, parents noted weaker fine motor skills. He had some friends at school but occasionally exhibited aggressive behaviour towards other children or took their toys. At that moment school-related concerns outweighed parental concerns.

#### 2.2.2. Methods

The boy underwent an assessment at a centre for developmental disorders (COS Leuven, Belgium) at the age of 3 to monitor his overall development and address behavioural issues at school, prompted by the identified KIF11 mutation. A comprehensive developmental screening, encompassing cognitive testing, play observation, and logopedic and motor examinations, conducted by a multidisciplinary team produced the following results (Table 1).

At the age of 4, the boy underwent a re-evaluation and follow-up assessment at COS Leuven after the initiation of therapy. Developmental examinations, including class observation, medical assessment, and motoric evaluation, were conducted. The diagnostic criteria outlined in the 5^th^ edition of the Diagnostic and Statistical Manual of Mental Disorders (DSM–5) serve as the framework for reaching a diagnosis of a developmental disorder such as ASD or ADHD [8].

Just over a year later, at the age of 5.5, a child psychiatrist conducted a diagnostic evaluation. This assessment encompassed child psychiatric evaluation, a comprehensive developmental history review, and the administration of the Autism Diagnostic Observation Schedule 2 (ADOS-2). The ADOS-2, one of the gold standards in diagnosing ASD, yielded a score of 11 (cut-off 9), providing ample evidence for an autism classification.

#### 2.2.3. Outcome and Follow-Up

The first assessment at COS Leuven, at the age of 3, concluded that the patient possesses average cognitive and language abilities, along with adequate gross motor skills. However, his fine motor skills appeared delayed. Impaired vision may have contributed to the behavioural challenges. Additionally, the boy demonstrated a strong seeking of stimuli, and (hyper)activity impeded his ability to focus, with early signs of ADHD noted. Socially, he exhibited some foundational skills but displayed certain autistic traits, including special interests, sensory oversensitivity, associative thoughts, and socially less attuned behaviour. The initiation of therapy, encompassing physiotherapy, home counselling, and individual behavioural programmes, was recommended, with a follow-up planned after 1 year.

Based on the re-evaluation at COS 1 year later, sufficient characteristics were identified to confirm the diagnosis of ADHD, including overexcitability, limited attention, and impulse control. This diagnosis was officially established by a child neurologist. Additionally, certain traits associated with ASD were noted, particularly in social interaction, such as lesser attunement, limited engagement with peers, and occasional seeking of (negative) attention. However, these characteristics were not pronounced enough to confirm the diagnosis, and a follow-up by a child psychiatrist was recommended to monitor these traits over time. After the diagnosis of ADHD was made, recommendations were provided for interventions. The boy had already attended physiotherapy for his motor skills, and it was suggested to continue with this treatment. Additionally, parents were advised to submit a request for home counselling to address the behavioural issues at home, particularly focusing on potty training. At the time of diagnosis, the boy was still enrolled in a regular primary school with additional support. Despite receiving daily assistance to make it manageable for both him and the teachers, his behaviour, work ethic, and overall well-being continued to decline. Consensus among parents and the school was reached to transition him to special needs education, as he became progressively more unhappy, aggressive, and resistant, with tasks not being completed due to the necessity for continual adjustments. Pharmacotherapy was initiated with methylphenidate at a dosage of 5 mg, twice daily, for the management of ADHD symptoms, such as impulsive behaviour and attention deficits.

Through the observations of the child psychiatrist, the diagnosis of ASD was confirmed. Upon the confirmation of an ASD diagnosis, the previously recommended interventions were maintained. Pharmacological treatment, specifically methylphenidate at a dosage of 5 mg twice daily, was also continued since it had a positive impact on ADHD symptoms.

As the child psychiatrist who initially diagnosed ASD didn't provide follow-up for patients older than 6, the parents had to seek out a new specialist. They commenced follow-up with a different child psychiatrist at the start of 2023. Currently, there are quarterly evaluations with this new psychiatrist, primarily focusing on medication management to address persistent emotional and behavioural issues. There have been six consultations with the new child psychiatrist so far (until July 2024).

Recently, at the age of 8, treatment was initiated with the antipsychotic aripiprazole at a dosage of 2.5 mg once a day to improve behavioural and emotional regulation. This decision was made because the boy exhibited increased irritability and more frequent and intense emotional outbursts and had difficulty listening to parents and teachers. The feedback from both parents and the school was positive, with the boy being less irritable, better at following instructions, and showing more mental flexibility. This medication was used in conjunction with methylphenidate at a dosage of 10 mg twice daily. The dosage of methylphenidate was doubled a few months after the start of aripiprazole because of the boy's increased weight and the reduced effectiveness on ADHD symptoms, such as the attention deficits over time.

At present, the boy is almost 9 years old and takes aripiprazole 5 mg once a day in combination with methylphenidate 10 mg twice daily, which has stabilised his emotional and behavioural regulation and controlled his ADHD symptoms at an acceptable level for now. Nevertheless, core ASD symptoms, including difficulties with social interactions with peers and his younger sister, remain a concern.

## 3. Discussion

Intellectual disabilities are frequently associated with KIF11 mutations, while other neurodevelopmental disorders such as ASD and ADHD are less commonly reported. Jones et al. documented cases of autism with ADHD in one patient and behavioural issues in another [6]. Malvezzi et al. [5] reported a single case of ADHD, and Mirzaa et al. [11] mentioned two cases of ADHD and two with aggressive behaviour.

In our case, it is noteworthy that two developmental disorders, namely, ASD and ADHD, occur without intellectual disability. Additionally, the mother of the boy mentioned that she has been in contact with other parents of children with KIF11 mutations, and many of these families reported that their children also have ASD and/or ADHD. Therefore, we designed a poll in a parent support group for children with KIF11 mutations in December 2023. The results can be found in Table 2. Sixty-three parents participated, revealing that 60% of their children diagnosed with KIF11-associated disorder had ASD, ADHD, or both, while the remaining 40% had no such diagnosis. Some in this latter group exhibited no developmental disorder features and were not examined, some were too young for testing, and a final subset underwent examination but lacked sufficient characteristics for a diagnosis. Globally, around 1% of the general population is diagnosed with ASD, and 5%–7% with ADHD (depending on age, higher in primary school age) [12, 13]. Despite the relatively small size of our group, the data strongly suggest a higher prevalence of neurodevelopmental disorders, ASD, and ADHD in the KIF11 group compared to the general population. The notable disparity between prevalence and available literature on this topic is remarkable. However, it's crucial to interpret these numbers cautiously, recognising that more diagnostic testing is likely in children with a KIF11 mutation, often due to associated intellectual disability.

Despite increased testing, children with a KIF11 mutation may experience delays in seeing a child psychiatrist. In Belgium, the diagnosis of ASD or ADHD can only be made by a (child) psychiatrist or a (child) neurologist, ideally within a multidisciplinary team setting. In our patient's case, the KIF11-associated disease was suspected in April 2017 and confirmed in January 2018, but the first consultation with a child psychiatrist occurred only in January 2019, more than 2.5 years after the KIF11 diagnosis was made. This delay was partly due to long waiting lists for child psychiatrists in Belgium [14]. The long waiting times represent a well-known issue that poses a significant challenge for healthcare in Belgium. As long as the problem persists, early referral remains the only way to provide patients with timely support.

It is noteworthy that children with a KIF11 mutation are regularly monitored by a paediatrician and an ophthalmologist but not by a child psychiatrist. However, establishing a strong collaboration with child psychiatry is essential. Children with a KIF11 mutation are now typically referred for child psychiatric diagnostics primarily due to behavioural problems or intellectual disability. We recommend that, when identifying a KIF11 mutation, the patient is followed by both an ophthalmologist experienced in the disease and a paediatrician for monitoring potential lymphoedema and other general problems. The paediatrician can make low-threshold referrals to a child psychiatrist when the potential presence of developmental problems is suspected. Early interventions and diagnostic testing by a child psychiatrist can significantly improve the patient's and their family's quality of life by addressing various areas of concern.

For children with ASD, addressing symptoms as early as possible enables parents to acquire effective strategies for enhancing their children's social and communication skills (e.g., through in-home counselling sessions) while also reducing the risk of more severe secondary symptoms [15]. It is known that the brain exhibits high levels of neuroplasticity during early childhood, making it more adaptable and responsive to these interventions. Furthermore in the Belgian school system, a diagnosis of a developmental disorder is required to gain access to the most appropriate type within special education. For example, children with ASD and normal intelligence could gain access to type 9 education, where specific attention is given to their developmental needs, including features like sensory-friendly spaces and small class sizes. For ADHD, specifically initiating methylphenidate treatment more promptly yields better long-term outcomes [16]. Overall, recent studies highlight that individuals with rare disorders in general confront challenges that extend beyond medical issues. Many of these difficulties could be mitigated through greater societal awareness and understanding of rare disorders, along with increased attention to psychological health and adaptive coping strategies [17]. This further emphasises the importance of considering the mental well-being of patients with any kind of rare disorder and not only in our specific case.

Furthermore, a better understanding of the increased prevalence of ASD and ADHD in individuals with KIF11 mutations could provide valuable insights into the genetic pathogenesis of neurodevelopmental disorders. This connection might reveal how variations in the KIF11 gene contribute to the development of these conditions, helping to elucidate the underlying mechanisms that influence neurodevelopment and potentially guiding more targeted approaches for diagnosis and treatment.

## 4. Conclusion

Our case involves an 8-year-old boy with a KIF11 mutation who has been diagnosed with ASD and ADHD. We advise early referral to child psychiatry for interventions with the purpose of improving quality of life for the patient and his family, as individuals with KIF11 cases may exhibit a higher prevalence of these neurodevelopmental disorders compared to the general population.

## Figures and Tables

**Table 1:** Results of developmental assessments.

Skills	Cognitive	Language	Motor
2019 (age: 3 years to 3 years 2 months)	SON-R 2 ½ - 7: TIQ 103 (3 years 2 months) • PS: 91 • LR: 114	RDLS: CS: 53/87107 pc. 68 (3; 06 y) PS: • Vocabulary: 22/28 pct. 85(4; 00 y • Language content: 14/39 pct. 30 (3; 00 y) • Spontaneous language: 13/22 pct. 60 (3; 06 y)	Bayley-III-NL: Fine motor skills: 413 (28 months) Gross motor skills: 628 (35 months)

2020 (age: 4 years to 4 years 1 month)	—	—	PDMS-2: Fine motor skills: 879 Gross motor skills: 1081 Grips: pct. 9 (3 years 1 month) Visuomotor integration: pct. 16 (3 years 4 month) Balance: pct. 9 (2 years 11 months) Locomotor skills: pct. 37 (3 years 10 months) Object manipulation: pct. 9 (2 years 9 months)

Abbreviations: Bayley-III-NL, Bayley scales of infant and toddler development III; CS, comprehension scale; IQPS, performance scale; IQRS, reasoning scale; Pct., percentile; PDMS-2, peabody developmental motor scales; PS, production scale; RDLS, Reynell developmental language scales; SON-R, Snijders-Oomen nonverbal intelligence test-Revised; TIQ, total intelligence quotient.

**Table 2:** Poll in parent support group about the prevalence of ASD and/or ADHD in patients with KIF11 mutation.

Developmental disorder	Prevalence (63 votes)
ASD	20% (12/63)
ADHD	25% (16/63)
Both (ASD + ADHD)	15% (9/63)
Neither	40% (26/63)

## Data Availability

The authors confirm that the data supporting the findings of this study are available within the article.
